# Culture Negative Infective Endocarditis Presenting as Renal Infarction in a Low-risk Young Woman: A Case Report

**DOI:** 10.31729/jnma.v64i293.9300

**Published:** 2026-01-31

**Authors:** Sandip Pandey, Anu Timalsina, Sagar Pokhrel, Vivek Chhetri, Nawaraj Ranabhat

**Affiliations:** 1 B.P. koirala Institute of Health Sciences, Dharan, Sunsari, Nepal; 2 Patan Academy of Health Sciences, Lagankhel, Lalitpur, Nepal

**Keywords:** *case report*, *embolism*, *infective endocarditis*, *renal infarction*, *right kidney*

## Abstract

Culture-negative infective endocarditis is difficult to diagnose because it often presents with vague symptoms, delaying recognition and worsening outcomes. Renal infarction is rare but may be the first clue. A previously healthy 32-year-old woman had intermittent fever for six weeks and sudden right-sided flank pain for two days. Examination revealed a holosystolic murmur and right costovertebral angle tenderness. Investigations showed raised inflammatory markers and a positive rheumatoid factor, while repeated blood cultures were negative. Echocardiography showed severe mitral regurgitation without visible vegetations, and computed tomography angiography confirmed right renal infarction. Using modified Duke criteria, the diagnosis was made and empiric intravenous vancomycin and ceftriaxone were started. Despite intensive care and aggressive therapy, she deteriorated and died from refractory heart failure due to severe valvular dysfunction. This case shows the disease can be insidious even in low-risk patients, stressing early suspicion, multidisciplinary care, and timely surgical evaluation when appropriate.

## INTRODUCTION

Infective endocarditis (IE) is a serious infection of the endocardial surface of the heart, most commonly affecting cardiac valves. Culture-negative infective endocarditis (CNIE) poses a particular diagnostic challenge, often resulting from prior antibiotic therapy or infection with fastidious organisms.^1,2^ In resource-limited regions such as South Asia, where rheumatic heart disease remains prevalent (1-5 per 1,000 individuals) and access to advanced diagnostic tools (transthoracic echocardiography, laboratory investigation) is limited, CNIE frequently goes unrecognized.^3^ It may present atypically, even without vegetations on echocardiography^4^. This case report highlights a rare (<1%) renal infarction presentation of CNIE in a young, low-risk (early-age, no prior history of cardiac disease, no prior antibiotic exposure/invasive dental procedure done) woman who developed renal infarction as an initial manifestation of systemic embolization.

## CASE REPORT

A 32-year-old woman with no prior comorbidities presented to the emergency department with a six-week history of intermittent fever and two days of acute right flank pain. The fever was undocumented, low-grade, and associated with malaise, while the flank pain was constant, dull, and accompanied by nausea and vomiting. She denied chills, hematuria, dysuria, joint pains, rashes, recent dental procedures, or any history of intravenous drug use. She was married and has two children, a 7-year-old daughter and a 4-year-old son. Her antenatal and postnatal periods were unremarkable. There was no personal or family history of autoimmune disease, hypercoagulable states, or prior valvular heart disease.

On examination, she was febrile (38.8°C) and pale, with a blood pressure of 110/70 mmHg. Cardiovascular assessment revealed a new grade 3/6 holosystolic murmur at the apex radiating to the axilla. Abdominal examination revealed tenderness over the right costovertebral angle. No Osler nodes, Janeway lesions, splinter hemorrhages, or Roth spots were identified.

Initial laboratory investigation revealed, significant anemia with a hemoglobin level of 9.1 g/dL, and leukocytosis (WBC 13,400/μL) with neutrophil predominance (78%), suggesting an ongoing inflammatory or infectious process. Inflammatory markers were markedly elevated, with an erythrocyte sedimentation rate of 108 mm/hr and C-reactive protein of 92 mg/L. Platelet count was within normal limits. Renal function was preserved (serum creatinine 0.9 mg/dL), and urinalysis was unremarkable despite radiologic evidence of renal infarction. An immunologic phenomenon was suggested by a positive rheumatoid factor (1:160), while autoimmune markers including ANA, antidsDNA, and ANCA were negative. Three sets of blood cultures remained negative, and extensive serologic testing for fastidious organisms including *Coxiella burnetii, Bartonella* spp., *Brucella* spp., and *Legionella* was negative, supporting the diagnosis of culture-negative infective endocarditis. Other than that, the USG showed normal findings at the time.

**Table 1:** Laboratory investigations on admission.

Test	Result	Reference Range
Hemoglobin	9.1 g/dL	12-16 g/dL
WBC count	13,400/μL	4,000-11,000/μL
Neutrophils	78%	40-70%
Platelets	280,000/μL	150,000-400,000/μL
ESR	108 mm/hr	<20 mm/hr
CRP	92 mg/L	<5 mg/L
Serum Creatinine	0.9 mg/dL	0.6-1.2 mg/dL
Rheumatoid Factor	Positive (1:160)	Negative
ANA	Negative	Negative
Anti-dsDNA	Negative	Negative
ANCA	Negative	Negative
Blood cultures × 3	Sterile	-
Serologies (*Coxiella, Bartonella, Brucella, Legionella*)	Negative	-
Urinalysis	Normal	-

Transthoracic and transesophageal echocardiography revealed thickened mitral valve leaflets with severe new mitral regurgitation, but no vegetations or abscesses were visualized (Figure 1a, 1b, 1c). CT angiography ofthe abdomen demonstrated a wedge-shaped infarction in the right kidney, consistent with an embolic phenomenon (Figure 2). Chest radiography showed mild cardiomegaly without pulmonary infiltrates.

Given the clinical presentation of fever, embolic renal infarction, and new mitral regurgitation, the Modified Duke Criteria were applied. The patient fulfilled one major criterion (new valvular regurgitation) and three minor criteria (fever ≥38°C, vascular phenomenon, renal infarction, and positive rheumatoid factor), confirming the diagnosis of definite culture-negative infective endocarditis.

**Figure 1a:**
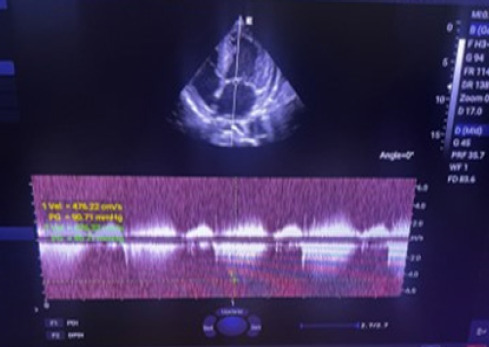
Continuous Wave doppler echocardiography tracing shows high velocity jet and elevated peak pressure gradient across mitral valve demonstrating severe MR.

**Figure 1b:**
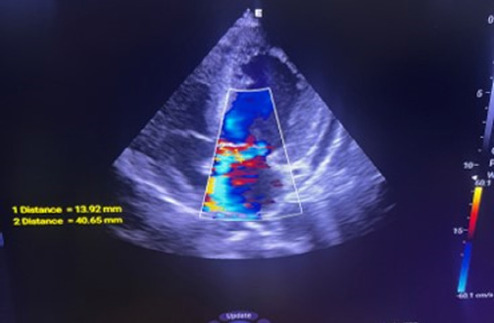
Regurgitant jet on color doppler echocardiography demonstrating mitral regurgitation.

**Figure 1c:**
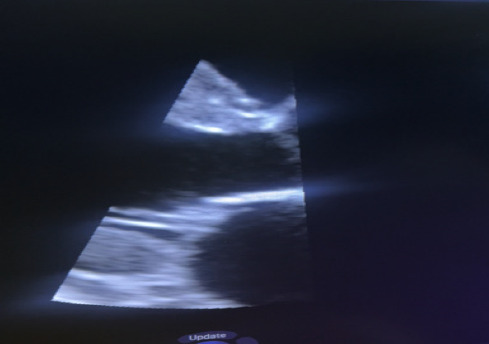
2D echocardiographic view of mitral valve demonstrating thickened mitral valve with no vegetation.

Differential diagnoses were systematically excluded. Systemic lupus erythematosus was ruled out by negative ANA and antidsDNA and the absence of clinical criteria. Rheumatic carditis was unlikely due to the absence of prior pharyngitis, migratory arthritis, and negative antistreptolysin O titers. Vasculitis was excluded based on normal urinalysis and negative ANCA testing. Hypercoagulable states were considered unlikely given normal coagulation profiles and the absence of thrombotic history. The patient had received multiple courses of oral antipyretics before reaching our centre but not been exposed to antibiotics. Empiric treatment with intravenous ceftriaxone (2 g daily) and doxycycline (100 mg twice daily) was initiated, targeting likely fastidious organisms. Anticoagulation was withheld due to the risk of hemorrhagic complications. Supportive care included hydration and antipyretics. Despite broad-spectrum antibiotic therapy, the patient’s clinical condition did not improve, and antibiotics were upgraded to vancomycin (1gm twice daily), necessitating multidisciplinary consultation (cardiology, infectious disease specialist, radiologist, cardiothoracic surgeon) for potential surgical intervention. Despite optimal medical therapy, the patient developed progressive heart failure and shock, requiring transfer to the intensive care unit for advanced support. Despite aggressive management, she succumbed to refractory heart failure secondary to severe mitral regurgitation on 14^th^ day of admission, and a post-mortem was not considered.

**Figure 2:**
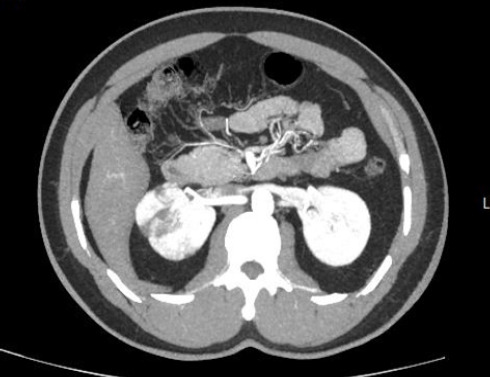
CT angiography revealed a wedge-shaped perfusion defect in the right kidney, consistent with renal infarction.

## DISCUSSION

This case report highlights the challenging diagnosis of culturenegative infective endocarditis in a young woman whose initial presentation was dominated by a renal infarction. The main findings were prolonged fever, negative blood cultures, a new heart murmur and evidence of embolization without vegetations on TTE, which shows the difficulty in diagnosing atypical infective endocarditis. The diagnosis was ultimately established based on the modified Duke criteria, where the presence of a major criterion (new valvular regurgitation) and multiple minor criteria (fever, vascular phenomena, immunologic) was met.^5^

A key limitation in this case is that transesophageal echocardiography (TEE) was not available. TEE is more sensitive than transthoracic echocardiography (TTE) for detecting small vegetations, abscesses, and prosthetic valve involvement^6^. The absence of TEE reduces the ability to definitively exclude small vegetations or paravalvular complications, which could have influenced both diagnostic certainty and management decisions. Despite this, the diagnosis was established using the Modified Duke Criteria based on new valvular regurgitation and supportive minor criteria^7^. Our findings are consistent with existing literature that describes the diagnostic difficulties of Culture-Negative Infective Endocarditis. The proportion of IE cases that are culture-negative can be as high as 35%, often due to prior antibiotic use or fastidious pathogens^8^. While TTE detects only 45% of vegetations seen on TEE, negative blood cultures are associated with negative TEE results^9^. However, a clearly negative TTE examination in patients with native valves and adequate image quality may be sufficient to rule out infective endocarditis, with a negative predictive value of 100% in one study^10^.

The strengths of this report include the comprehensive diagnostic workup, which successfully identified a renal infarct as the embolic source and confirmed severe valvular damage despite the absence of vegetations. This underscores the principle that embolic events are a critical diagnostic clue for IE. A limitation of this report is the inability to identify a specific causative organism due to the lack of advanced molecular or serological testing, which could have provided a definitive microbiological diagnosis and guided targeted therapy. Such tests are not always readily available, particularly in resource-limited settings.

This case has significant implications for clinical practice. It reinforces the need for clinicians to maintain a high index of suspicion for IE in any patient presenting with prolonged fever and evidence of arterial embolization, even if blood cultures are negative and no vegetations are seen on echocardiography. For health policy, it highlights the importance of developing standardized protocols for CNIE that include early access to advanced diagnostic tools like PCR and serology. From a research perspective, there is a clear need for studies aimed at identifying novel biomarkers that can facilitate the early diagnosis of CNIE and for clinical trials to determine the optimal timing of surgical intervention in patients with severe valvular destruction who do not respond to empiric antibiotic therapy. Unlike most reported cases of CNIE, our patient had no underlying cardiac risk factors or prior antibiotic exposure, underscoring the need for vigilance even in apparently low-risk individuals.

## CONCLUSION

This case of culture-negative infective endocarditis presenting as renal infarction in a young, otherwise healthy woman underscores the deceptive nature and diagnostic complexity of this condition. The absence of positive blood cultures or echocardiographic vegetations should not exclude the diagnosis when clinical and imaging findings are suggestive. Clinicians should maintain a high index of suspicion for CNIE in patients with prolonged fever and embolic phenomena, even in the absence of definitive microbiological confirmation. Early recognition, multidisciplinary collaboration, and timely surgical consideration are essential to improving outcomes and reducing mortality in such challenging cases.
